# Pseudopheochromocytoma With Catecholamine Excess and End-organ Damage: A 30-year Course Treated With Escitalopram

**DOI:** 10.1210/jcemcr/luaf321

**Published:** 2026-01-27

**Authors:** Ayli S Anvaripour, Leor Needleman, Brian Brady, Justin P Annes

**Affiliations:** Indiana University School of Medicine, Indianapolis, IN 46202, USA; Department of Medicine, Division of Endocrinology, Gerontology and Metabolism and Endocrine Oncology, Stanford University, Stanford, CA 94305, USA; Department of Medicine, Division of Nephrology, Stanford University School of Medicine, Stanord, CA 94305, USA; Department of Medicine, Division of Endocrinology, Gerontology and Metabolism and Endocrine Oncology, Stanford University, Stanford, CA 94305, USA

**Keywords:** Pseudopheochromocytoma, paroxysmal hypertension, catecholamine excess, serotonin reuptake inhibitor

## Abstract

Pseudopheochromocytoma is a disorder characterized by paroxysmal hypertension and variably elevated catecholamine metabolite levels. Pseudopheochromocytoma clinically mimics pheochromocytoma but differs in etiology. While pheochromocytoma is a catecholamine-secreting neuroendocrine tumor, pseudopheochromocytoma is a syndrome linked to a history of emotional stressors and is believed to stem from autonomic nervous system dysregulation. We present the case of a 70-year-old female patient experiencing episodic hypertensive crises for 3 decades. The patient was referred to endocrine oncology for evaluation of a possible pheochromocytoma due to her long-standing history of symptomatic hypertension and elevated catecholamine metabolites. Anatomic and functional imaging, including computed tomography scans of the abdomen and pelvis and a ^64^Copper-1,4,7,10-tetraazacyclododecane-1,4,7,10-tetraacetic acid-octreotate positron emission tomography computed tomography excluded a diagnosis of pheochromocytoma or paraganglioma. Her history of significant emotional stressors raised the possibility of pseudopheochromocytoma. Following initiation of escitalopram and psychotherapy, the patient experienced a remarkable improvement in the frequency and severity of hypertensive episodes. This case illustrates the diagnostic challenges of pseudopheochromocytoma and the importance of early intervention in preventing complications.

## Introduction

Pseudopheochromocytoma closely mirrors the clinical manifestations of pheochromocytoma with symptoms of flushing, headache, diaphoresis, and palpitations [1]. Pseudopheochromocytoma is characterized by sudden increases in blood pressure that often occur in the absence of an acute emotional trigger. The hypertensive episodes can lead to hospitalizations and end-organ damage [2]. Pseudopheochromocytoma is a psychosomatic disorder believed to arise from disturbances in the autonomic nervous system. It is often associated with a history of abuse or profound psychological distress [1, 2]. It is suspected that many cases remain undiagnosed due to prominent physical symptoms and limited patient insight into contributing psychological factors [3]. Although the pathogenesis of pseudopheochromocytoma remains unclear, studies have identified elevated baseline plasma epinephrine or metanephrine levels in some patients and an exaggerated physiological response to endogenous catecholamines in others [1, 4]. Pseudopheochromocytoma is considered a diagnosis of exclusion, necessitating evaluation for the differential diagnoses including pheochromocytoma, functioning paragangliomas, carcinoid tumors, hyperaldosteronism, renovascular disease, obstructive sleep apnea (OSA), hyperthyroidism, panic disorder, posttraumatic stress disorder, medication withdrawal, and illicit substance use [1, 5].

Per Mann's diagnostic criteria, pseudopheochromocytoma is diagnosed when abrupt, paroxysmal hypertensive episodes with adrenergic symptoms occur without an emotional trigger and in the absence of any evidence of adrenal or extra-adrenal tumors [2]. Here, we report a case involving a 70-year-old female who presented with life-threatening hypertensive crises and a biochemical profile compatible with catecholamine-secreting neuroendocrine tumor. After neoplastic and alternative causes of her presentation were excluded, the patient met the diagnostic criteria for pseudopheochromocytoma. She was treated with psychotherapy and escitalopram and experienced a marked reduction in hypertensive episodes.

## Case Presentation

A 70-year-old Caucasian female and retired emergency department (ED) nurse received a referral to endocrinology for evaluation of a possible pheochromocytoma due to her long-standing history of episodic hypertension. The patient reported that hypertensive episodes and symptoms were first noted in her 40s and progressively increased in frequency and severity during the past 3 decades. In her 40s, she began to experience episodic systolic blood pressures reaching 140 to 170 mmHg with associated symptoms of flushing and headache. She was treated with various blood pressure medications at that time. At the age of 59, the patient experienced a hypertensive episode with systolic blood pressure reaching 220 mmHg, requiring intensive care unit admission for 5 days. Clonidine was identified as an effective treatment. At the age of 62, the patient reported that she began to experience episodic diarrhea, altered mentation, and elevated lactic acid levels. These episodes required intensive care unit-level care and occurred every 5 to 6 months; 1 episode resulted in a transient ischemic attack and another in pulmonary edema.

At the time of presentation to our institution (age 70), the patient reported experiencing 2 to 3 hypertensive episodes per month over the preceding 6 months. She stated that hypertensive episodes were typically preceded by diaphoresis, diarrhea, and vomiting. She denied preceding psychological distress as a trigger for her symptoms. She noted that the episodes were triggered by bowel movements but were not associated with large meals. She denied use of tobacco, alcohol, or recreational substances. Medical history was significant for obstructive sleep apnea managed with continuous positive airway pressure therapy and cyclic vomiting syndrome diagnosed during adolescence. The patient was on several blood pressure medications and reported adding hydralazine or nifedipine for hypertensive episodes. Prochlorperazine and promethazine were prescribed as needed for nausea and vomiting occurring during hypertensive paroxysms. The comprehensive list of medications is given in Table 1.

**Table 1.** Patient's medication list at the time of initial presentation to endocrinology, including antihypertensive medications, antiemetics, and other therapies

Medication	Dosage and administration	Frequency
Clonidine	0.3 mg, transdermal patch 0.2 mg, PO	Weekly patch, 3 PO doses daily
Nebivolol	2.5 mg, PO	Twice daily
Sacubitril-Valsartan	24 mg/26 mg, PO	Twice daily
Amlodipine	5 mg, PO	Once daily at bedtime
Empagliflozin	10 mg, PO	Once daily
Hydralazine	10 mg, PO	Every 8 hours PRN
Nifedipine ER	60 mg, PO	Once daily PRN
Ondansetron	4 mg, sublingual	Every 8 hours PRN
Prochlorperazine	10 mg, PO	Every 6 hours PRN
Prochlorperazine	25 mg suppository, rectal	Every 12 hours PRN
Promethazine	25 mg suppository, rectal	At bedtime PRN

Abbreviations: PO, *per os* (by mouth); PRN, *pro re nata* (as needed).

When asked about adverse life events, the patient described an emotionally taxing career as a nurse. She reported relying on emotional suppression and managing distressing experiences by mentally placing them into an imagined file drawer, which she would never revisit. She was initially evaluated through a virtual visit. She appeared well and in no acute distress, with no abnormal skin findings. Her body mass index was 31 kg/m^2^.

## Diagnostic Assessment

The patient's most recent ED visit for hypertensive crisis (3 months prior to her initial endocrinology evaluation), in the context of long-standing paroxysmal hypertension, prompted consideration of pheochromocytoma. Toxicology screening obtained in the ED yielded negative results. Laboratory testing revealed elevated urine catecholamine metabolites, with urine metanephrine 407 µg/24 hours (SI: 2.06 µmol/24 hours) (reference: 36-229 µg/24 hours [SI: 0.18-1.16 µmol/24]) and urine normetanephrine 1751 µg/24 hours (SI: 9.56 µmol/24 hours) (reference: 95-650 µg/24 hours [SI: 0.52-3.56 µmol/24 hours]). The patient's initial laboratory results are summarized in Table 2.

**Table 2.** Initial screening for pheochromocytoma demonstrating abnormally elevated urine normetanephrine, metanephrine, norepinephrine, and epinephrine concentrations

Test	Result	Reference range
Urine normetanephrine	1751 µg/24 hours (9.56 µmol/day)	95-650 µg/24 hours (0.52-3.56 µmol/day)
Urine metanephrine	407 µg/24 hours (2.06 µmol/day)	36-229 µg/24 hours (0.18-1.16 µmol/day)
Urine norepinephrine	190 µg/24 hours (1.12 µmol/day)	14-120 µg/24 hours (0.08-0.71 µmol/day)
Urine epinephrine	38 µg/24 hours (0.21 µmol/day)	1-14 µg/24 hours (0.005-0.077 µmol/day)
Urine dopamine	416 µg/24 hours (2.71 µmol/day)	71-485 µg/24 hours (0.46-3.16 µmol/day)

Values in parentheses are in Système International units.

Further diagnostic evaluation including assessment of pituitary hormone axes and 5-hydroxyindoleacetic acid, as well as a comprehensive metabolic panel yielded normal results. Plasma renin activity and plasma aldosterone concentration were both suppressed on repeat outpatient testing, potentially reflecting physiologic suppression of the renin-angiotensin-aldosterone system in the setting of hypertension. Alternatively, this pattern may reflect the physiology of pseudopheochromocytoma or the effects of her medications including nebivolol, valsartan, and clonidine, which are known to lower renin and aldosterone [6]. Laboratory findings are summarized in Table 3 and Table 4. Plasma and urine catecholamine metabolites were normal on repeat outpatient testing performed 3 months after the initial elevation (Table 5).

**Table 3.** Results of the comprehensive metabolic panel and endocrine workup, showing normal electrolytes, BUN, creatinine, glucose, AST, ALT, urine 5-HIAA, and low aldosterone and plasma renin activity

Test	Result	Reference range
Sodium	141 mmol/L	135-146 mmol/L
Potassium	4.0 mmol/L	3.5-5.3 mmol/L
Chloride	106 mmol/L	98-110 mmol/L
CO_2_	27 mmol/L	20-32 mmol/L
Urea nitrogen	11 mg/dL (3.9 mmol/L)	7-25 mg/dL (2.5-8.9 mmol/L)
Creatinine	0.93 mg/dL (82.3 µmol/L)	0.60-1.00 mg/dL (53-88 µmol/L)
Calcium	9.6 mg/dL (2.39 mmol/L)	8.6-10.4 mg/dL (2.15-2.60 mmol/L)
Glucose	85 mg/dL (4.7 mmol/L)	65-99 mg/dL (3.6-5.5 mmol/L)
Albumin	4.2 g/dL (42 g/L)	3.6-5.1 g/dL (36-51 g/L)
Bilirubin	1.0 mg/dL (17.1 µmol/L)	0.2-1.2 mg/dL (3.4-20.5 µmol/L)
AST	19 U/L	10-35 U/L
ALT	15 U/L	6-29 U/L
ALP	73 U/L	37-153 U/L
Total serum protein	7.0 g/dL	6.1-8.1 g/dL
Plasma renin activity, LC-MS/MS	0.10 ng/mL/h (0.10 µg/L/h)	0.25-5.82 ng/mL/h (0.25-5.82 µg/L/h)
Aldosterone, serum	2 ng/dL (55.5 pmol/L)	Upright AM ≤ 28 ng/dL (≤778 pmol/L)
Cortisol, urine	31.3 µg/24 h (86.3 nmol/day)	4.0-50.0 µg/24 h (11-138 nmol/day)
5-HIAA, urine	4.8 mg/24 h (25.2 µmol/day)	<6.0 mg/24 h (<31.4 µmol/day)

Values in parentheses are in Système International units.

Abbreviations: 5-HIAA, 5-hydroxyindoleacetic acid; ALP, alkaline phosphatase; ALT, alanine transaminase; AST, aspartate transaminase; LC-MS/MS, liquid chromatography–tandem mass spectrometry.

**Table 4.** Patient's pituitary evaluation revealing normal ACTH, cortisol, TSH, free T4, and IGF-1 levels

Test	Result	Reference range
ACTH	9 pg/mL (2.0 pmol/L)	6-50 pg/mL (1.32-11.0 pmol/L)
Cortisol	6.9 µg/dL (190 nmol/L)	4.0-22.0 µg/dL (110-600 nmol/L)
TSH	1.05 mIU/L	0.40-4.50 mIU/L
Free T4	1.2 ng/dL (15.5 pmol/L)	0.8-1.8 ng/dL (10-23 pmol/L)
IGF-1	53 ng/mL (6.9 Nmol/L)	41-279 ng/mL 5.4-36.6 Nmol/L

**Table 5.** Results of the repeat biochemical evaluation for pheochromocytoma, including urine metanephrines, catecholamines, and plasma metanephrines, all within normal limits

Test	Result	Reference range
Metanephrine 24-hour, urine	221 µg/24 hours (1.12 µmol/day)	36-229 µg/24 hours (0.18-1.16 µmol/day)
Normetanephrine 24-hour, urine	441 µg/24 hours	95-650 µg/24 hours
	(2.41 µmol/day)	(0.52-3.55 µmol/day)
Dopamine 24-hour, urine	212 µg/24 hours (1.38 µmol/day)	52-480 µg/24 hours (0.34-3.12 µmol/day)
Norepinephrine 24-hour, urine	12 µg/24 hours (71 nmol/day)	15-100 µg/24 hours (89-591 nmol/day)
Epinephrine 24-hour, urine	<3 µg/24 hours (<16.4 nmol/day)	2-24 µg/24 hours (11-131 nmol/day)
Calculated total catecholamines, urine	12 µg/24 hours (65.4 nmol/day)	26-121 µg/24 hours (142-662 nmol/day)
Normetanephrine, plasma	50 pg/mL (0.27 nmol/L)	≤148 pg/mL (≤0.80 nmol/L)
Metanephrine, plasma	<25 pg/mL (<0.13 nmol/L)	≤57 pg/mL (≤0.30 nmol/L)

Values in parentheses are in Système International units.

**Figure 1.**
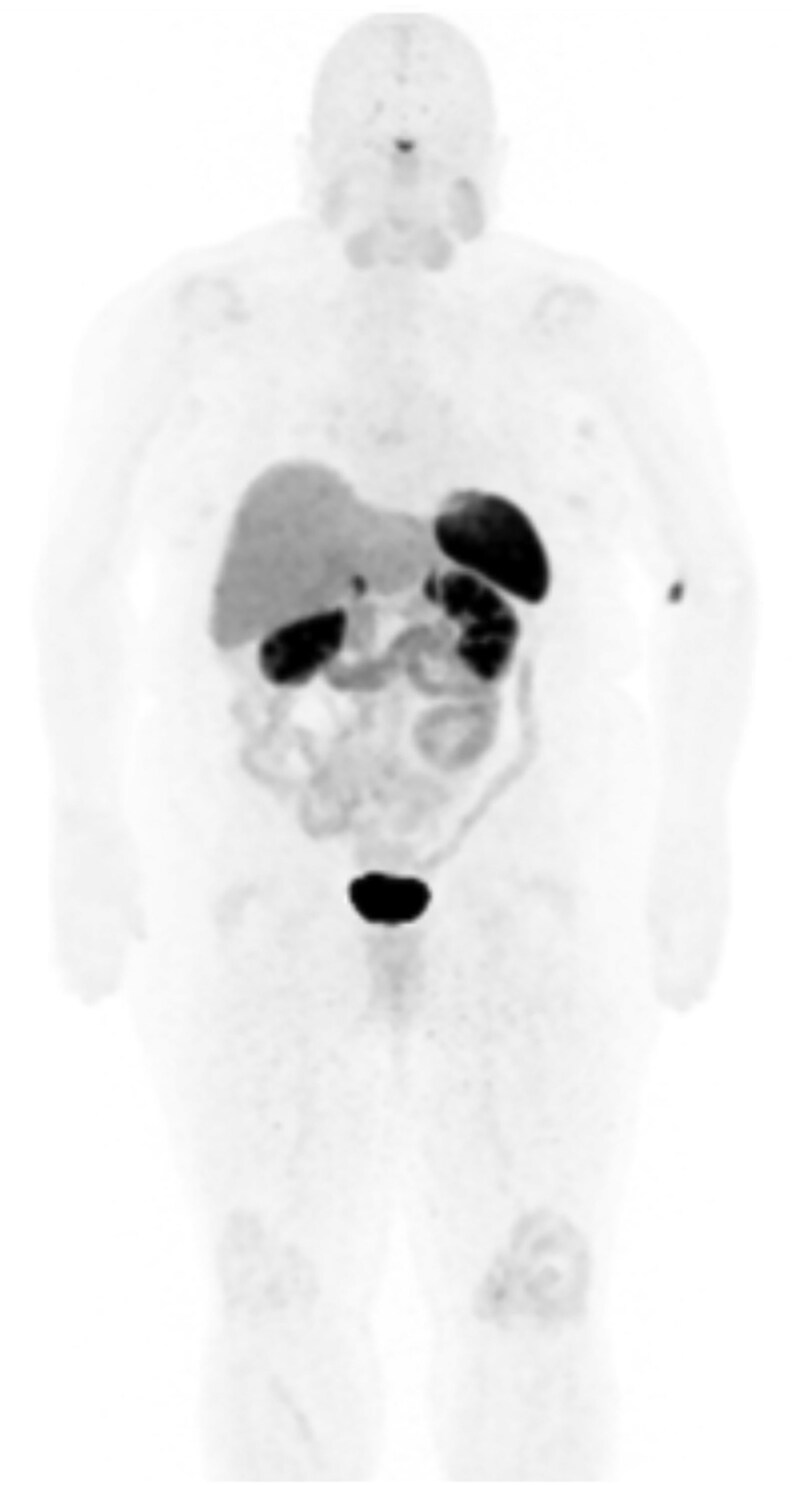
Cu-64 DOTATATE PET/CT maximum intensity projection did not reveal any sites of abnormal radiotracer uptake. Abbreviation: Cu-64 DOTATATE PET-CT, ^64^Copper-1,4,7,10-tetraazacyclododecane-1,4,7,10-tetraacetic acid-octreotate positron emission tomography computed tomography.

The patient underwent a brain magnetic resonance imaging to evaluate for central nervous system injury related to hypertensive crisis, which was negative for infarct or hemorrhage but incidentally revealed a partially empty sella. She completed a renal duplex ultrasound that was negative for renal artery stenosis. Computed tomography of the abdomen and pelvis did not reveal any adrenal abnormalities, and ^64^Copper-1,4,7,10-tetraazacyclododecane-1,4,7,10-tetraacetic acid-octreotate positron emission tomography computed tomography was negative for adrenal or extra-adrenal masses, effectively excluding pheochromocytoma or paraganglioma (Fig. 1).

The patient completed a transthoracic echocardiogram, which was notable for left ventricular hypertrophy. Given her history of emotional stressors and a negative workup for neuroendocrine tumors, the diagnostic criteria proposed by Mann were applied to support the diagnosis of pseudopheochromocytoma (Table 6).

**Table 6.** Key clinical features of pseudopheochromocytoma [2]

Clinical features	Description
Hypertensive paroxysms	Sudden onset of hypertensive episodes
Physical symptoms	Chest pain, headache, diaphoresis, nausea, palpitations, flushing, dyspnea, weakness
Trigger pattern	Episodes typically not provoked by emotional distress or panic
Psychiatric history	History of severe trauma or abuse

## Treatment

The patient was started on 20 mg of escitalopram oxalate taken orally once daily and referred to psychiatry.

## Outcome and Follow-up

Seven months after initiating treatment, the patient reported only experiencing 2 hypertensive episodes during that period and stated that she felt better than she had in decades.

These hypertensive episodes were approximately a week apart and began with bloating, diarrhea, and vomiting. The medication regimen was simplified to clonidine 0.3 mg transdermal patch applied once weekly and hydralazine taken orally every 8 hours as needed for hypertensive episodes. Over the subsequent 18 months, she has experienced no hypertensive admissions and only infrequent, mild elevations in blood pressure.

## Discussion

The term *pseudopheochromocytoma* was used by Kuchel in 1985 to describe paroxysmal hypertension with suspected autonomic dysfunction [7]. Historically, pseudopheochromocytoma has been predominantly diagnosed in women [3]. Although the pathogenesis of pseudopheochromocytoma remains unclear, it is believed that prior trauma or abuse may contribute to heightened sympathetic nervous system activity, resulting in symptom manifestation [4].

Fewer than 2% of patients with episodic hypertension and suggestive symptoms are diagnosed with pheochromocytoma [8]. However, due to the potential for complications and comorbidities associated with a missed diagnosis, pheochromocytoma remains a frequent focus of screening. The diagnostic evaluation of pheochromocytoma begins with biochemical testing for confirmation of elevated catecholamine metabolite levels, followed by imaging studies for tumor localization [1]. Markedly elevated catecholamine metabolites strongly suggest pheochromocytoma; however, more modest increases do not exclude it, as catecholamine secretion may be episodic or localized, or the tumor burden may be low [9, 10].

Patients with pseudopheochromocytoma generally exhibit normal plasma and urine catecholamine metabolite levels; however, transient elevations during paroxysms have been documented [1]. Although our patient had elevated urine metanephrine and normetanephrine levels, repeat biochemical testing and imaging studies ruled out pheochromocytoma or paraganglioma. Several additional factors could have potentially contributed to the elevated catecholamine levels that were initially observed. Although hospitalization-related stress may have contributed to these elevations, the patient's presentation with classic paroxysmal symptoms suggests a pathologic surge rather than nonspecific stress. Possible prochlorperazine use, prior to presentation, may have contributed to transiently increased catecholamine metabolites; however, this pharmacologic effect of dopamine receptor antagonists is primarily observed when a functional pheochromocytoma or paraganglioma is present [11]. False-positive increases in catecholamine metabolite levels are more strongly associated with the use of norepinephrine reuptake-blocking medications [12]. The patient's catecholamine levels were normal on the same medication regimen, making medication usage alone an unlikely explanation for the marked elevation in catecholamine metabolites levels.

Case reports have suggested that OSA may, in some instances, present as paroxysmal hypertension [13]. Since the patient's OSA was managed with appropriate continuous positive airway pressure use, OSA was unlikely to be the cause of her presentation. The literature on pseudopheochromocytoma describes a range of adverse life experiences, including physical and emotional abuse [14, 15]. However, psychological stress is not limited to abuse-related trauma, and other significant stressors as observed in our patient may also contribute to autonomic dysregulation.

Treatment approaches for pseudopheochromocytoma include antihypertensives, antidepressants, anxiolytics, cognitive behavioral therapy, and behavioral interventions such as meditation. In 1999, Mann demonstrated that most patients with pseudopheochromocytoma who received a combination of antidepressants, α- and β-adrenoceptor blockers, and psychotherapy experienced a reduction in hypertensive episodes [3]. In a more recent study, treatment with sertraline was associated with symptom improvement in 75% of patients with pseudopheochromocytoma [16]. α- and β-adrenergic blockers may aid in controlling hypertensive paroxysms in some patients by attenuating an exaggerated physiological response to catecholamines. Severe episodes typically require hospitalization and IV agents such as sodium nitroprusside or nicardipine [1]. Conventional antihypertensive medications that do not target adrenergic pathways are often ineffective in the treatment of pseudopheochromocytoma, and selective serotonin reuptake inhibitors are essential for long-term management [1]. Pseudopheochromocytoma can mimic a multitude of functional tumor and nonneoplastic syndromes, leading to a prolonged and costly diagnostic process. The case presented in this report illustrates the challenge of diagnosing this underrecognized condition.

## Learning Points

- This case illustrates the diagnostic challenges of pseudopheochromocytoma, in part due to the variability in catecholamine metabolite levels.
- Considering psychological factors in patients with paroxysmal hypertension may facilitate earlier diagnosis, reduce costly hospitalizations, and lower the risk of associated complications.
- The combined use of selective serotonin reuptake inhibition and psychotherapy has apparent efficacy in the management of patients with pseudopheochromocytoma.

## Contributors

All authors made individual contributions to authorship. J.P.A., L.N., and B.B. were involved in the diagnosis and management of this patient and manuscript revisions. A.S.A. made substantial contributions to the interpretation and integration of the patient's clinical presentation and drafted all sections of the manuscript. All authors reviewed and approved the final draft.

## Data Availability

Data sharing is not applicable to this article as no datasets were generated or analyzed during the current study.
